# CREBH Systemically Regulates Lipid Metabolism by Modulating and Integrating Cellular Functions

**DOI:** 10.3390/nu13093204

**Published:** 2021-09-15

**Authors:** Yoshimi Nakagawa, Masaya Araki, Song-iee Han, Yuhei Mizunoe, Hitoshi Shimano

**Affiliations:** 1 Department of Complex Biosystem Research, Institute of Natural Medicine, University of Toyama, Toyama 930-0194, Toyama, Japan; 2 Department of Endocrinology and Metabolism, Faculty of Medicine, University of Tsukuba, Tsukuba 305-8575, Ibaraki, Japan; s1930432@s.tsukuba.ac.jp (M.A.); shan@md.tsukuba.ac.jp (S.-i.H.); ymizunoe@md.tsukuba.ac.jp (Y.M.); 3 International Institute for Integrative Sleep Medicine (WPI-IIIS), University of Tsukuba, Tsukuba 305-8575, Ibaraki, Japan; 4 Life Science Center for Survival Dynamics, Tsukuba Advanced Research Alliance (TARA), University of Tsukuba, Tsukuba 305-8577, Ibaraki, Japan; 5 Japan Agency for Medical Research and Development–Core Research for Evolutional Science and Technology (AMED-CREST), Chiyoda-ku, Tokyo 100-0004, Japan

**Keywords:** CREBH, SREBP, lipid metabolism, fatty liver, atherosclerosis, gene expression

## Abstract

Cyclic AMP-responsive element-binding protein H (CREBH, encoded by CREB3L3) is a membrane-bound transcriptional factor expressed in the liver and small intestine. The activity of CREBH is regulated not only at the transcriptional level but also at the posttranslational level. CREBH governs triglyceride metabolism in the liver by controlling gene expression, with effects including the oxidation of fatty acids, lipophagy, and the expression of apolipoproteins related to the lipoprotein lipase activation and suppression of lipogenesis. The activation and functions of CREBH are controlled in response to the circadian rhythm. On the other hand, intestinal CREBH downregulates the absorption of lipids from the diet. CREBH deficiency in mice leads to severe hypertriglyceridemia and fatty liver in the fasted state and while feeding a high-fat diet. Therefore, when crossing CREBH knockout (KO) mice with an atherosclerosis model, low-density lipoprotein receptor KO mice, these mice exhibit severe atherosclerosis. This phenotype is seen in both liver- and small intestine-specific CREBH KO mice, suggesting that CREBH controls lipid homeostasis in an enterohepatic interaction. This review highlights that CREBH has a crucial role in systemic lipid homeostasis to integrate cellular functions related to lipid metabolism.

## 1. Introduction

Cyclic AMP-responsive element-binding protein 3-like 3 (*CREB3L3*, encoding the CREBH protein) is expressed in only the liver and small intestine. CREBH, an endoplasmic reticulum (ER) membrane-bound transcription factor, is controlled by regulated intramembrane proteolysis (RIP). RIP is a posttranslational process that cleaves transmembrane precursor proteins to release them from the cell membrane. CREBH is localized in the ER and is transported to the Golgi apparatus. The transmembrane domain of the CREBH protein is cleaved by the site-1 and site-2 proteases in the Golgi apparatus, producing the active form of the CREBH protein and subsequently transporting it to the nucleus [1]. CREBH contains a b-Zip domain, a hydrophobic stretch, a leucine zipper domain, and a KDEL-like sequence (an ER retrieval sequence). CREBH has a high homology with the cAMP response element-binding protein (CREB)/Activating transcription factor (ATF) family of molecules via its b-Zip domain. LZIP (Luman/CREB3), a member of the CREB/ATF family, is a homolog of box-B binding factor-2 (BBF-2). BBF-2 binds not only to the box-B element but also to the cAMP response element (CRE) sequence. CREBH binds to both the CRE and box-B sequences in the promoter region to activate gene expression

**Figure 1:**
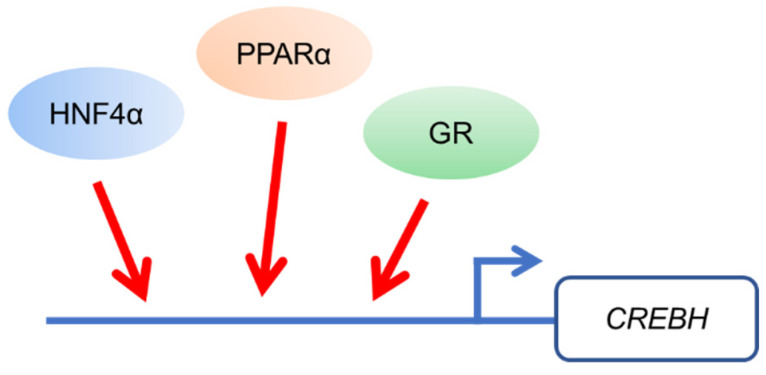
Gene expression of Cyclic AMP-responsive element-binding protein H (CREBH) is up-regulated by transcription factors including peroxisome proliferator-activated receptor α (PPARα), hepatocyte nuclear factor 4α (HNF4α), and glucocorticoid receptor (GR) during fasting.

Hepatic *CrebH* mRNA is apparently increased by peroxisome proliferator-activated receptor α (PPARα) [2], hepatocyte nuclear factor 4α (HNF4α) [3], and glucocorticoid receptor (GR) [4] during fasting (Figure 1). Subsequently, CREBH controls fasting-induced gene expression in the liver and maintains nutrient condition to adapt to fasting. Thus, CREBH deficiency leads to various metabolic disorders, such as fatty liver, hyperlipidemia, and atherosclerosis. 

## 2. CREBH and PPARα Synergistically Control Lipid Metabolism

In a fasted state, plasma glucose and insulin levels are low, which stimulates lipolysis to increase fatty acids in the white adipose tissue (WAT) [5,6]. Subsequent increased fatty acid levels in the blood are incorporated into the liver. In the liver, the incorporated fatty acids are resolved through fatty acid β-oxidation, which converts fatty acids into acetyl coenzyme A (Acetyl-CoA [7]). Acetyl-CoA subsequently synthesizes ketone bodies as an alternative energy source to glucose [8]. In a fasted state, as triglyceride synthesis is inhibited in the liver, liver metabolism is lipolytic. However, when the levels of fatty acids exceed the lipolytic capacity in the liver, excess fatty acids are stored in the liver as triglyceride. Thus, hepatic triglyceride levels are increased in the fasted state. PPARα is a key modulator that controls gene expression related to fatty acid β-oxidation as a transcription factor in hepatic adaptation to fasting [9]. Thus, PPARα knockout (KO) mice show a deficiency of lipid metabolism and subsequent severe fatty liver in the fasted state [9].

CREBH is a modulator of PPARα to increase the gene expression of *Ppara* and its target genes. In a fasted state, CREBH KO mice exhibit a significant reduction in *Ppara* expression. Conversely, PPARα KO mice exhibit a reduction in *CrebH* expression. CREBH and PPARα reciprocally regulate each other’s gene expression and transcriptional activity in a fasted state. Thus, deficiency of CREBH in mice shows a remarkable reduction in these transcription factors’ target genes, resulting in severe fatty liver similar to PPARα KO mice [10,11]. Thus, as the changes in the gene expression in the liver of CREBH KO and PPARα KO mice overlap, it is difficult to identify their direct target genes. By comparing the gene expression in the liver between CREBH KO mice and PPARα KO mice in a fasted state, the direct targets of CREBH have been identified as carnitine palmitoyltransferase 1a, liver (*Cpt1a*) in fatty acid oxidation and D-β-hydroxybutryate dehydrogenase (*Bdh1*) in ketogenesis [11]. When a PPARα agonist such as fenofibrate is administrated in CREBH KO mice, fenofibrate-mediated PPARα activation is completely diminished, indicating that CREBH is essential to fully activate PPARα function [10].

**Figure 2:**
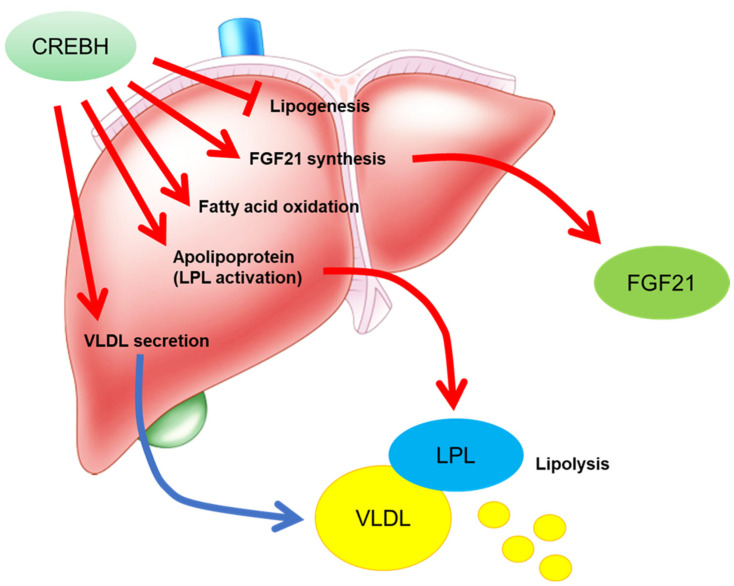
The functions of CREBH in the liver. CREBH inhibits lipogenesis by suppressing sterol-regulatory element-binding protein (SREBP) activation. CREBH activates fibroblast growth factor 21 (FGF21) expression, subsequent increasing FGF21 synthesis and secretion. FGF21 improves lipid metabolism in the peripheral tissues. CREBH directly and indirectly activates PPARα function to increase its target gene expression. CREBH modulates gene expression of apolipoprotein to activate lipoprotein lipase (LPL). CREBH increases very low-density lipoprotein (VLDL) secretion to regulated to its related gene expression in VLDL secretion. Taken together, CREBH efficiently reduces plasma triglyceride levels to activate lipolysis.

CREBH transgenic mice show significantly increased hepatic gene expression and plasma levels of plasma fibroblast growth factor 21 (FGF21) (Figure 2) [10,12]. FGF21 is an FGF family member, but it is secreted from the liver into the blood. Hepatic FGF21 expression is induced by fasting or feeding a ketogenic diet [13,14]. Conversely, in CREBH KO mice, fasting-induced hepatic FGF21 expression is reduced [10]. CREBH directly binds to the FGF21 promoter and upregulates FGF21 expression [10,15]. PPARα also directly upregulates FGF21 expression in the liver in response to fasting [13,14]. The binding sites of CREBH and PPARα on the FGF21 promoter are close to each other, and both factors synergistically activate FGF21 expression. CREBH supports the formation of a complex between PPARα and PPARɤ coactivator-1α (PGC-1α), thereby activating FGF21 expression.

FGF21 normalizes plasma glucose, insulin, and triglyceride levels in both ob/ob and db/db mice, two type 2 diabetes mouse models [16]. FGF21 induces thermogenic gene expression and browning in the WAT [17], but FGF21 KO mice have impaired adaptation to cold exposure with a defect in the browning of WAT [17]. FGF21 regulates this process by increasing PGC-1α protein levels in WAT [17]. The increase in the hepatic gene expression and plasma levels of FGF21 by CREBH overexpression activates energy expenditure with an increase in thermogenesis gene expression, including genes such as uncoupling protein 1 (*Ucp1*) and peroxisome proliferator-activated receptor gamma coactivator 1α (*Ppargc1a*), in the WAT [10].

## 3. CREBH Controls Fasting-Induced Lipophagy

Autophagy, a cellular catabolic process in the lysosomes, degrades cytoplasmic components, abnormally accumulated proteins, and damaged organelles. Autophagy plays a crucial role in maintaining cellular energy homeostasis and mobilizing nutrients under starvation or chronic metabolic stress conditions [18,19,20]. Autophagy is required for the adaptation to starvation [21] and is a catabolic process that supplies energy by recycling cytoplasmic components including damaged organelles and proteins under starvation [22,23]. The autophagy-mediated degradation of intracellular lipids has been termed lipophagy and supplies energy in starvation by providing free fatty acids for fatty acid β-oxidation and ATP production [18]. Transcription factor EB (TFEB) and Transcription factor E3 (TFE3), which are members of the MiT-TFE family of transcription factors, are crucial regulators of autophagy and lysosomal biogenesis that control gene expression related to these processes [24,25]. TFEB and TFE3 improve hyperlipidemia and diabetes [26,27,28], but it is unclear if these effects are mediated by autophagy. In normal conditions, TFEB is localized in the cytosol, but fasting induces the translocation of TFEB to the nucleus [25]. TFEB gene expression is activated during starvation through a positive feedback loop with PPARα and PGC-1α, and TFEB controls gene transcription related to lipid catabolism [27]. TFEB regulates gene expression related to the transport of fatty acid chains across the plasma membrane and β-oxidation. Thus, PPARα and TFEB control autophagy and lipophagy in the fasted state [27,29]. FGF21 is induced by PPARα during fasting [13,14] and autophagy deficiency [30]. FGF21 promotes TFEB target gene expression. Taken together, PPARα, PGC-1α, FGF21, and TFEB form a feed-forward loop and control lipid metabolism by regulating, for example, lysosome biogenesis, autophagy, and lipid oxidation. As mentioned above, CREBH is closely related to the PPARα-FGF21 axis, and CREBH controls autophagy and lysosomal biogenesis [31]. CREBH increases the gene expression of key enzymes and regulators in autophagosome formation and autophagic processes, including microtubule-associated protein light chain 3 (*Lc3*), autophagy-related protein 7 (*Atg7*), *Atg2b*, and unc-51-like kinase 1 (*Ulk1*), and genes related to lysosomal biogenesis and homeostasis [31]. CREBH regulates *Tfeb* expression in cooperation with PPARα and PGC-1α [31]. Thus, CREBH deficiency exacerbates fatty liver during fasting because of not only dysfunction of fatty acid oxidation but also dysfunction of autophagy.

## 4. Deficiency of CREBH Exacerbates Diet-Induced Steatohepatitis

On feeding a nonalcoholic fatty liver disease (NAFLD)-inducing diet, CREBH KO mice exhibit exacerbation of NAFLD and non-alcoholic steatohepatitis (NASH). When fed an atherogenic high-fat diet, CREBH KO mice exhibit severe accumulation of hepatic lipid metabolites and an increase in plasma triglyceride levels. CREBH controls hepatic gene expression related to triglyceride synthesis, cholesterol synthesis, fatty acid elongation, fatty acid oxidation, lipolysis, lipolysis-stimulated lipoprotein receptor, and lipid transport [32].

Feeding high-fat high-sucrose (HFHS) diet efficiently induces obesity and diabetes. Mice overexpressing the active form of CREBH in the liver (CREBH Tg mice) suppress both HFHS diet-induced obesity [10,12]. The gene expression of FGF21 is activated in the liver of CREBH Tg mice, activating systemic energy expenditure [10,12]. FGF21 increases browning of WAT and BAT, leading to increased energy expenditure [17]. CREBH Tg mice increase the gene expression related to thermogenesis, *Ucp1* and *Cidea*, and mitochondria function, elongase of very long chain fatty acids-3 (*Elovl3*) in the iWAT, activating browning [12]. As a result, CREBH Tg mice exhibit the suppression of diet-induced obesity (DIO) and fatty liver [12]. However, even if CREBH Tg mice are crossed with FGF21 KO mice, anti-DIO effects by CREBH overexpression still remains [12]. Thus, CREBH suppresses diet-induced fatty liver by FGF21-dependent and -independent mechanisms. However, FGF21-independent effect is not well unknown. 

When CREBH KO mice are fed a high-fat low-carbohydrate diet (ketogenic diet; KD), these mice exhibit severe fatty liver [33]. KD feeding is the condition to use fatty acids as a major energy source such as fasting. KD-fed CREBH KO mice activate hormone sensitive lipase (HSL) and adipose triglyceride lipase (ATGL) in the WAT compared with the WT mice by reducing FGF21 production from the liver, indicating that CREBH deficiency dysregulates adipose tissue lipolysis [33]. The increase of fatty acid flow from adipose tissue to the liver promotes fat accumulation in CREBH KO mice. KD-fed CREBH KO mice also reduce hepatic gene expression of PPARα and its target genes [33], thereby exacerbating fatty liver. FGF21 overexpression in CREBH KO mice ameliorate hepatosteatosis, by inhibiting lipolysis in the WAT with the reduction of HSL and ATGL activity [33]. These findings indicate that CRBEH-FGF21 improves hepatosteatosis by suppressing adipose tissue lipolysis. 

When fed a methionine- and choline-deficient (MCD) diet, which is another NASH-inducing model, CREBH KO mice exhibit severe liver fibrosis accompanied by higher plasma alanine transaminase (ALT) and hepatic hydroxyproline levels [34]. Hepatic gene expression related to inflammation and fibrosis, including chemokine (C-C motif) ligand 2 (*Ccl2*); actin alpha 2, smooth muscle, aorta (*Acta2*); desmin (*Des*); collagen, type I, alpha 1 (*Col1a1*); tissue inhibitor of metalloproteinase 1 (*Timp1*); transforming growth factor beta 1 (*Tgfb1*); and *Tgfb2*, was increased in CREBH KO mice. Deficiency of CREBH might primarily control the TGF-β1 signaling pathway, resulting in more severe inflammation and fibrosis [34]. Nakagawa et al. developed CREBH flox mice using a one-step CRISPR/Cas9 system and then generated liver-specific CREBH KO (CREBH LKO) mice [35]. When fed an MCD diet, CREBH LKO mice exhibited severe hepatitis without an increase in lipid levels in the liver [35]. The levels of plasma liver injury markers, ALT and aspartate transaminase, are severely increased in CREBH KO mice. Furthermore, the gene expression of inflammation and liver fibrosis-related genes were significantly increased in the livers of CREBH LKO mice [35]. Taken together, deficiency of CREBH in the liver contributes to the development of NAFLD and NASH.

## 5. CREBH Regulates Atherosclerosis Development by Controlling Lipid Metabolism in Enterohepatic Interactions

**Figure 3:**
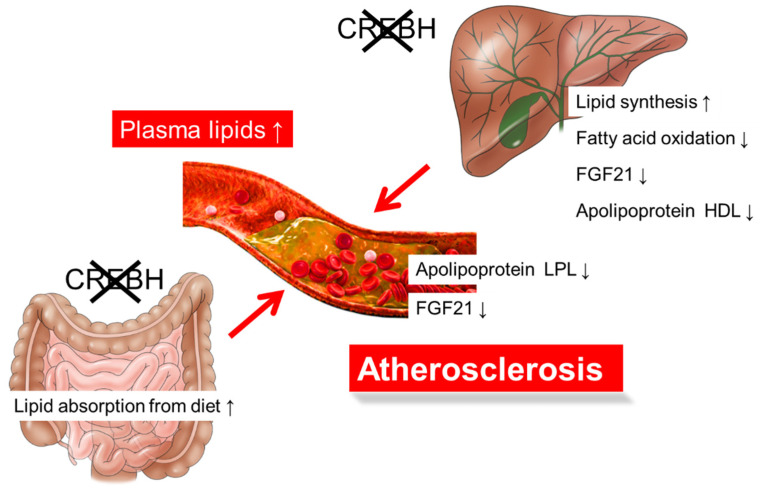
The metabolic dysfunctions in CREBH deficiency in low-density lipoprotein receptor knockout (LDLR KO) mice exacerbate atherosclerosis. Deficiency of CREBH in the liver exhibits the activation of lipid synthesis, inactivation of fatty acid oxidation and FGF21 expression, and abnormal function of apolipoprotein metabolism, increasing plasma lipid levels. Deficiency of CREBH in the small intestine increases lipid absorption from diet, increasing plasma lipid levels. Metabolic dysfunction due to CREBH deficiency in both the liver and small intestine of CREBH KO:LDLR KO mice synergistically increases plasma lipid levels and accelerate atherosclerosis development. ↑, level increased; ↓, level decreased.

Atherogenic dyslipidemia is characterized by high plasma triglyceride and low-density lipoprotein (LDL) levels and low plasma high-density lipoprotein (HDL) levels, which are risk factors for the development of atherosclerosis. Lipoprotein lipase (LPL)-mediated triglyceride clearance is important for the suppression of atherosclerosis [36]. Apolipoprotein A1 (ApoA1) is produced in the liver and small intestine and is the predominant component of HDL [37]. ApoA1 activates cholesterol efflux from the peripheral tissues for reverse cholesterol transport by interacting with the ATP-binding cassette transporter A1 [38,39]. CREBH deficiency reduces *Apoa1* expression in both the liver and intestine, subsequently reducing plasma ApoA1 and HDL-cholesterol (HDL-C) levels [40]. CREBH and HNF4α cooperate to activate *Apoa1* expression [40]. ApoA4 regulates hepatic lipid levels by activating very low-density lipoprotein (VLDL) particle expansion and triglyceride efflux without increasing ApoB-Containing lipoprotein particles secreted from the liver [41,42,43]. Apoa4 is also a key molecule in HDL metabolism by activating lecithin: cholesterol acyltransferase (LCAT). LCAT is an enzyme that transfers cholesterol to newly synthesized HDL particles to convert free cholesterol into cholesteryl esters [44,45], which activates cholesterol efflux from macrophages [46] and the receptor-mediated uptake of HDL by hepatocytes [47]. *Apoa4* transgenic mice show protection against atherosclerosis [48,49,50]. CREBH directly upregulates *Apoa4* expression in the liver and small intestine [51]. CREBH deficiency in LDL receptor (LDLR) KO mice exhibit high VLDL-triglyceride and low HDL-C levels in the plasma and accelerated atherosclerosis. In contrast, CREBH overexpression in the liver reduces plasma triglyceride levels. CREBH overexpression in LDLR KO mice increases apolipoprotein-related genes, such as *Apoa1*, *Apoa4*, *Apoa5*, and *Apoc2*, which stimulates LPL-mediated triglyceride clearance (Figure 2 and Figure 3) [40,52]. Conversely, deficiency of CREBH in LDLR KO mice reduces the expression of these genes, thereby inducing hypertriglyceridemia [40,52].

FGF21 deficiency in ApoE KO mice results in severe atherogenic phenotypes [53], but administering FGF21 to these ApoE KO mice ameliorates atherosclerosis [54]. Thus, the exacerbation of atherosclerosis in CREBH KO mice might contribute to the dysfunction of the CREBH-FGF21 pathway. However, the overexpression of CREBH in FGF21 KO:LDLR KO mice improves atherosclerosis [52], suggesting that the contribution of FGF21 to the CREBH-mediated improvement of atherosclerosis is not substantial. Nonetheless, the contribution of FGF21 to arteriosclerosis is important. Therefore, further analysis is needed. Consistent with a previous report [55], the absorption of cholesterol from food in the small intestine is increased in CREBH KO:LDLR KO mice [52]. These changes contribute to an increase in plasma cholesterol levels, resulting in the acceleration of atherosclerosis development (Figure 3). Although CREBH is expressed in the liver and small intestine, the underlying question of in which organ CREBH is important for lipid metabolism had not been clarified because tissue-specific KO mice had not been generated. Nakagawa et al. examined this question using tissue-specific CREBH KO mice by crossing with LDLR KO mice [52]. Liver- or intestine-specific CREBH KO:LDLR KO exacerbated atherosclerosis [52], indicating that CREBH in both the liver and small intestine contributes to the pathology of atherosclerosis. Deficiency of CREBH in both the liver and small intestine exacerbated arteriosclerosis more than deficiency in each tissue alone [52]. Therefore, CREBH is likely to be important in both tissues.

**Figure 4:**
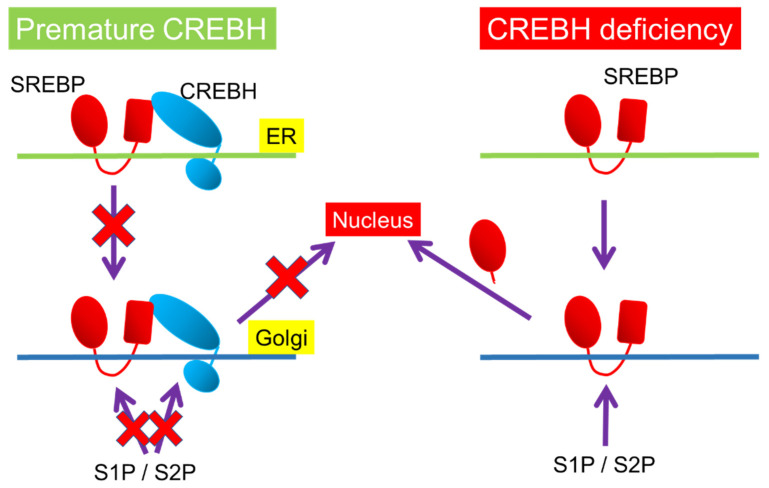
Post-translational mechanism in CREBH-SREBP interaction. Both premature forms of CREBH and SREBP reciprocally inhibits the transportation from the endoplasmic reticulum (ER) to the Golgi and cleavage by site-1 protease (S1P) and site-2 protease (S2P) in the Golgi. Premature CREBH inhibits SREBP cleavage, while CREBH deficiency induces it.

Sterol-regulatory element-binding proteins (SREBPs) are also membrane-bound transcription factors including SREBP-1a, -1c, and -2 to regulate the biosynthesis and uptake of lipids [56]. SREBP-1a regulates the gene expression related to the production of phospholipids, fatty acids, and cholesterol [56]. SREBP-1c regulates the gene expression related to the synthesis of fatty acids and triglycerides in response to overnutrition [56]. SREBP-2 maintain cholesterol levels to control the gene expression of the cholesterol synthesis-related genes in response to the demands on cellular sterols [56]. SREBPs and CREBH share the same posttranslational activation system, RIP. Both factors are localized in the ER and are transported to the Golgi, where they are cleaved by site-1 and site-2 proteases (S1P and S2P) (Figure 4). CREBH is activated by energy shortage, while SREBPs are activated by overnutrition. CREBH and SREBPs balance to regulate lipid metabolism at the transcriptional level. Cleaved factors are transported into the nucleus. SREBP cleavage-activating protein (SCAP) binds to SREBP and promotes the transport of SREBP from the ER to the Golgi. Insulin-induced gene 1 (Insig-1) and 2 (Insig-2) form a complex with SREBP-SCAP, inhibiting SREBP transport to the Golgi [57,58,59]. CREBH directly induces *Insig-2a* mRNA, a liver-specific isoform of Insig-2, in the liver, which downregulates the translocation of SREBP-1c and reduces *de novo* lipogenesis [60]. CREBH inhibits hepatic *de novo* lipogenesis via the induction of Insig-2a, thereby preventing hepatic steatosis and hypertriglyceridemia [60]. CREBH downregulates *de novo* lipogenesis, while SREBP activates it (Figure 2), thus indicating that the functions and activation conditions of CREBH and SREBP are complementary. If premature SREBP and CREBH coexist in the ER membrane, both factors physically interact with each other. This association reciprocally inhibits the transport from ER to Golgi each other (Figure 4) [52]. Consistent with this observation, deficiency of CREBH in the liver increased the level of nuclear SREBP, while deficiency of SREBP-1 in the liver increased the level of nuclear CREBH [52]. However, the overexpression of the nuclear form of CREBH does not change SREBP-target gene expression in the liver [52]. CREBH suppresses SREBP activation by intermolecular interaction and insig-mediated regulation.

## 6. CREBH Cooperates with Transcription Factors Related to Lipid Metabolism via the Circadian Rhythm

CREBH is regulated by the circadian clock via proteolytic cleavage, posttranslational acetylation modification, and protein degradation. CREBH is phosphorylated by glycogen synthase kinase 3β, modulating the association between CREBH and coat protein complex II transport vesicles, including Sec23, Sec24, and secretion associated Ras related GTPase 1 (Sar1). These associations are essential to transport CREBH from the ER to the Golgi, in which CREBH is cleaved and activated. This process is controlled by the circadian rhythm [61].

During the circadian rhythm, CREBH is associated with transcription factors such as PPARα and liver × receptor α (LXRα). The association of CREBH with PPARα or LXRα enhances CREBH transcriptional activity [62]. The acetylation of CREBH in response to the circadian rhythm is consistent with transcriptional activation of CREBH. The association between CREBH and PPARα is controlled via the circadian rhythm, but defects in the acetylation of CREBH block this association [62]. Moreover, the CREBH and PPARα interaction affects the control of CREBH protein stability. ER-associated degradation (ERAD) is an ER quality control mechanism that eliminates misfolded proteins by ubiquitination and proteasomal degradation [63,64]. ERAD controls CREBH by 3-hydroxy-3-methylglutaryl-CoA reductase degradation 1 homolog (Hrd1) and Suppressor/Enhancer of Lin-12-like (Sel1L). HRD1 is an E3 ubiquitin ligase, and SEL1L is an essential cofactor for HRD1 [65,66]. Hrd1/Sel1L controls the protein degradation of CREBH in the circadian rhythm, while CREBH cooperates with PPARα to directly regulate both *Hrd1* and *Sel1l* expression [67]. These molecules form a feedback loop with reciprocal regulation between Hrd1/Sel1L and CREBH [67]. Thus, defects in the Hrd1/Sel1L system lead to the activation of the CREBH-FGF21 pathway [67,68,69]. Liver-specific *Hrd1* KO mice show increased CREBH and FGF21 in the liver, resulting in growth retardation, female infertility, and impaired diurnal circadian behavior [67,68,69].

## 7. CREBH Links Starvation and Growth Delay

Starvation suppresses growth hormone (GH) signaling and inhibits growth, but the underlying mechanisms remain unknown. FGF21 causes GH resistance [70]. In the liver, FGF21 downregulates the signal transducer and activator of transcription 5 (STAT5), a major mediator of GH action, resulting in decreases in the gene expression of insulin-like growth factor 1 (IGF-1), a factor involved in body growth [70]. FGF21 induces the hepatic expression of IGF-1 binding protein 1 (*Igfbp1*) and the suppressor of cytokine signaling 2 (*Socs2*). These changes lead to the suppression of GH signaling. Thus, the FGF21-mediated suppression of GH signaling inhibits growth in mice [70], suggesting that this is an adaptive response to starvation. Similar to FGF21, hepatic CREBH transgenic mice have inhibited growth. CREBH liver-specific transgenic mice exhibit severe GH resistance [71]. Furthermore, *Fgf21* transgenic mice have a defect in the GH signaling protein STAT5, but liver-specific CREBH transgenic mice have a deficiency in GH receptor, an upstream molecule of STAT5 [71]. Thus, these phenotypes remain when crossing liver-specific CREBH transgenic mice with FGF21 KO mice [71]. Liver specific Hrd1 KO, another mouse model with elevated CREBH and FGF21 in the liver, causes the suppression of GH signaling and growth delay [68,69]. Thus, CREBH-FGF21 are crucial mediators linking fasting and growth delay.

## 8. Conclusions

Hepatic CREBH directly and indirectly controls gene expression related to lipid metabolism, resulting in the maintenance of hepatic lipid metabolism. Intestinal CREBH controls the expression of genes related to lipid absorption. Taken together, CREBH participates in enterohepatic interactions to maintain systemic lipid metabolism. CREBH regulates the activity of transcription factors related to lipid metabolism as a cofactor. Importantly, CREBH and PPARα reciprocally regulate the gene expression of each other, and CREBH is required for PPARα activation. The gene expression levels of *CrebH* and Ppara are controlled by the circadian clock, and both factors control the protein degradation of CREBH by directly inducing HRD1 and SEL1L. CREBH and PPARα synergistically activate the expression of PPARα target genes related to lipid metabolism, improving hyperlipidemia. Thus, CREBH transgenic mice have improved metabolic diseases, including diabetes, obesity, hyperlipidemia, and atherosclerosis. Conversely, CREBH KO mice have exacerbated severe fatty liver and atherosclerosis. However, the molecular mechanism of CREBH in controlling nutrient metabolism remains unknown. Further study of the interactions between CREBH and other transcription factors in enterohepatic association is important for understanding the mechanisms underlying CREBH as a regulator of nutrient metabolism.

## Data Availability

Not applicable.
